# The FPR2-induced rise in cytosolic calcium in human neutrophils relies on an emptying of intracellular calcium stores and is inhibited by a gelsolin-derived PIP_2_-binding peptide

**DOI:** 10.1186/1471-2121-11-52

**Published:** 2010-07-06

**Authors:** Huamei Forsman, Claes Dahlgren

**Affiliations:** 1 Department of Rheumatology and Inflammation Research, University of Gothenburg, Sweden

## Abstract

**Background:**

The molecular basis for neutrophil recognition of chemotactic peptides is their binding to specific G-protein-coupled cell surface receptors (GPCRs). Human neutrophils express two pattern recognition GPCRs, FPR1 and FPR2, which belong to the family of formyl peptide receptors. The high degree of homology between these two receptors suggests that they share many functional and signal transduction properties, although they exhibit some differences with respect to signaling. The aims of this study were to determine whether FPR2 triggers a unique signal that allows direct influx of extracellular calcium without the emptying of intracellular calcium stores, and whether the gelsolin-derived PIP_2_-binding peptide, PBP10, selectively inhibits FPR2-mediated transient rise in intracellular Ca^2+^.

**Results:**

The transient rise in intracellular Ca^2+ ^induced by agonists for FPR1 or FPR2 in human neutrophils occurred also in the presence of a chelator of Ca^2+ ^(EGTA). PBP10 inhibited not only FPR2-induced oxidase activity, but also the transient rise in intracellular Ca^2+^.

**Conclusions:**

Ca^2+ ^signaling mediated *via *FPR2 follows the same route as FPR1, which involves initial emptying of the intracellular stores. PBP10 inhibits selectively the signals generated by FPR2, both with respect to NADPH-oxidase activity and the transient rise in intracellular Ca^2+ ^induced by agonist exposure.

## Background

Neutrophil granulocytes are crucial for the outcome of the "battle" between the innate immune system and invading micro-organisms, and are key cells in the damaged tissues at sites of infection and inflammation. Neutrophil responses to endogenous and exogenous chemoattractants include locomotory responses, up-regulation of adhesion molecules, secretion of granule constituents, and production of reactive oxygen species (ROS), which are generated by the electron-transporting NADPH-oxidase system [1-3]. The molecular basis for cellular recognition of chemoattractants is their binding to specific cell surface receptors [4-8]. Despite the structural variability of the numerous extracellular ligands, many of them bind to (and activate) specific receptors belonging to a large family of pertussis toxin-sensitive, G-protein-coupled receptors (GPCRs). These receptors share a high degree of amino acid sequence similarity, and although they are activated by different agonists, they transduce downstream signals that have many common features. Nevertheless, it is clear that there are also important differences between the receptor-ligand pairs in terms of functional repertoires [9,10]. The pattern recognition formyl peptide receptor (FPR) family belongs to the GPCR group of chemoattractant receptors, and human neutrophil granulocytes express two members of this family, i.e., FPR1 and FPR2 [4,11]. FPR2 was originally defined as an orphan receptor, and the gene was cloned from an HL-60 cell cDNA library by low-stringency hybridization with the *FPR1 *sequence [12-14]. Recently, several FPR2-specific ligands have been identified [4,11], including mitochondrial and microbial peptides [15,16], various antimicrobial peptides [17], the acute phase protein serum amyloid A (SAA) [18,19], the neurotoxic prion peptide fragment 106-126 [20], and synthetic peptides, such as WKYMVM [21] and MMK-1 [22]. To date, no defined structure has been identified as the determinant for FPR2 binding and activation, although the close relationship between structural variation and function is illustrated by the fact that exchange of the C-terminal L-methionine residue in WKYMVM for the D-isomeric form expands the binding specificity to encompass both FPR2 and FPR1 [23].

The many studies that have been performed on FPR1-induced cell functions and signaling reveal that FPR1 signaling has all the characteristics of a pertussis toxin-sensitive GPCR. The activated receptor initiates a chain of signaling events, starting with dissociation of the receptor-associated G-protein, and subsequently, activation of a number of downstream signaling pathways. In one of these pathways, activation of phosphoinositide-specific phospholipase C (PLC) generates a second messenger following cleavage of PIP_2_, and this is the starting signal for a transient increase in cytosolic free calcium. Binding of the cleavage product, IP_3_, to its receptor located on storage organelles results in the release of Ca^2+ ^from these intracellular organelles and elevation consequent increase in the concentration of free calcium ions in the cytoplasm [Ca^2+^]_i _[24]. Emptying of the storage organelles leads to the entry of extracellular Ca^2+ ^through store-operated calcium channels in the plasma membrane, thereby prolonging the increase in [Ca^2+^]_i _[25,26].

Although our knowledge of the signal transduction pathways utilized by FPR2 is currently somewhat limited, the significant homology observed between FPR1 and FPR2 (69% at the amino acid level) suggests that these two receptors share signal transduction features. Accordingly, we have previously shown that the functional responses induced by the FPR2-specific agonist WKYMVM is largely similar to (even indistinguishable from) those induced by the prototype FPR1 agonist fMLF [4]. However, fundamental differences between the signaling profiles of these two receptors have been described; the PIP_2_-binding peptide PBP10 [27] selectively inhibits a signaling pathway triggered by FPR2, without affecting signaling *via *FPR1 [28]. FPR2 has also been shown to trigger a unique type of Ca^2+ ^influx across the plasma membrane [29]. It has been suggested that a channel in the plasma membrane opens without involvement of the intracellular storage organelles. Thus, the influx of Ca^2+ ^across the plasma membrane is not preceded by an increase in [Ca^2+^]_i _that originates from the release of Ca^2+ ^from the intracellular stores [29]. These results point to two important differences in the signaling mediated by the two FPRs in human neutrophils. Whereas FPR2 triggers a unique calcium signal, which is independent of the intracellular Ca^2+ ^store-influenced Ca^2+ ^channels, and allows for the direct influx of extracellular Ca^2+^, a PIP_2_-binding peptide inhibits FPR2-induced (but not FPR1-induced) radical production by neutrophils. It is noteworthy that the influx of calcium induced by FPR2 is apparently insensitive to PBP10 treatment [28]. The mechanism underlying these differences is puzzling and it is thus of importance to verify or falsify the observations.

In the present study, we characterize the neutrophil responses to the FPR2-specific agonist WKYMVM. We confirm the difference between FPR1-dependent and FPR2-dependent activation of the neutrophil NADPH-oxidase. However, in apparent discrepancy with previously published results [28,29], we show that the FPR2 agonist induces an increase in [Ca^2+^]_i _that involves Ca^2+ ^release from intracellular stores, and this signaling pathway is inhibited by the FPR2-specific inhibitor PBP10.

## Results

### The fMLF and WKYMVM peptides activate the NADPH-oxidase in neutrophils

**Figure 1:**
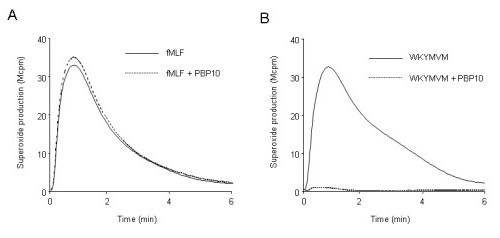
**Neutrophil release of superoxide anions induced by WKYMVM and fMLF, which are agonists for FPR2 and FPR1, respectively**. Neutrophils were stimulated with fMLF (A; 10^-7 ^M final concentration) or WKYMVM (B; 10^-7 ^M final concentration) in the absence or presence of PBP10 (A and B; 10^-6 ^M final concentration). The agonists were added at time 0, and the release of superoxide was followed using a chemiluminescence technique. Light emission was measured with (broken lines) or without (solid lines) the addition of PBP10. One representative of at least 100 experiments performed is shown. *Abscissa*, time of study (min); *ordinate*, superoxide production, given as light emission and expressed as cpm × 10^-6^.

The NADPH-oxidase activities induced by the FPR1-specific peptide agonist fMLF and the FPR2-specific peptide agonist WKYMVM were of similar magnitude (Fig. 1). The kinetics of the responses induced by equimolar concentrations (10^-7 ^M final concentrations) of these peptides were also very similar, with peak activity observed after approximately 1 minute. In accordance with earlier findings [28], the WKYMVM-induced response was inhibited by the membrane permeable polyphosphoinositide-binding peptide rhodamine-B-QRLFQVKGRR (PBP10), when it was added prior to activation. The neutrophil NADPH-oxidase activity was completely inhibited by PBP10 when activation was triggered through FPR2 (i.e., with WKYMVM), whereas PBP10 had no inhibitory effect on the FPR1-mediated (i.e., fMLF-triggered) neutrophil response (Fig 1). A molar ratio of agonist to inhibitor of 1:10 was required to inhibit the NADPH-oxidase response. Despite the very similar activation patterns of FPR1 and FPR2, functional differences between these two highly homologous receptors become apparent in the presence of PBP10.

### The fMLF and WKYMVM peptides mobilize intracellular Ca^2+ ^in neutrophils

**Figure 2:**
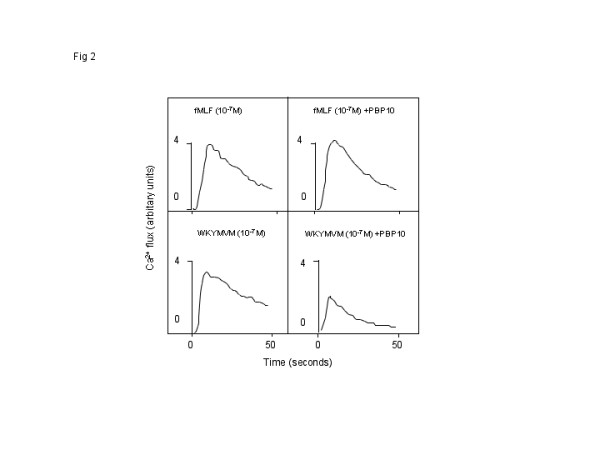
**The fMLF and WKYMVM peptides induce increases in intracellular calcium in neutrophils**. Neutrophils loaded with Fura-2 were incubated at 37°C for 5 minutes. PBP10 (10^-6 ^M final concentration) was present for the initial 5 minutes (B and D), and the cells were stimulated with fMLF (A and B; 10^-7 ^M final concentration) or WKYMVM (C and D; 10^-7 ^M final concentration). The changes in cytosolic Ca^2+ ^levels were determined by measuring the fluorescence emitted at 509 nm following excitation at 340 nm and 380 nm. The results are presented as the ratios of the fluorescence intensities at 340 nm and 380 nm, and a representative experiment (of at least five repetitions) is shown.

It is well known that binding of a specific ligand to FPR1 results in PLC-dependent cleavage of PIP_2_, and that the product, IP_3_, mobilizes calcium from intracellular storage organelles. Thus, a transient increase in intracellular free calcium was achieved when neutrophils were treated with the FPR1 agonist fMLF (Fig 2). Moreover, a similar response was obtained when neutrophils were treated with the FPR2-specific ligand WKYMVM (Fig 2). In the presence of extracellular calcium, the response is composed of two phases: 1) an initial phase that is dependent upon the release of Ca^2+ ^from intracellular stores; and 2) a second phase that is regulated by the stores [30] but relies on the opening of plasma membrane channels.

### The PIP_2_-binding peptide PBP10 inhibits the neutrophil calcium response to the FPR2 agonist WKYMVM

**Figure 3:**
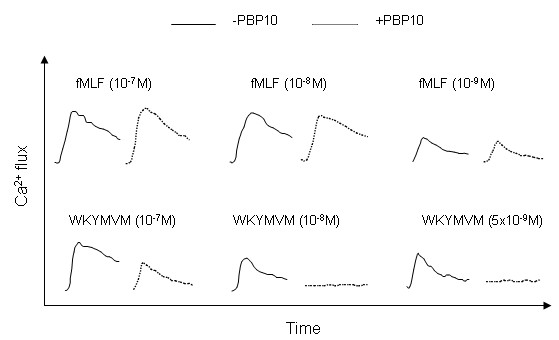
**The rise in intracellular calcium induced by low concentrations of WKYMVM in neutrophils is inhibited by PBP10**. Neutrophils loaded with Fura-2 were incubated at 37°C for 5 minutes. PBP10 (10^-6 ^M final concentration, broken lines) was present for the initial 5 minutes (control cells were incubated without PBP10, solid lines), and the cells were stimulated with decreasing concentrations of fMLF (upper part of the figure) or WKYMVM (lower part of the figure). The changes in cytosolic Ca^2+ ^levels were determined by measuring the fluorescence emitted at 509 nm following excitation at 340 nm and 380 nm. A representative experiment of at least five performed is shown.

**Figure 4:**
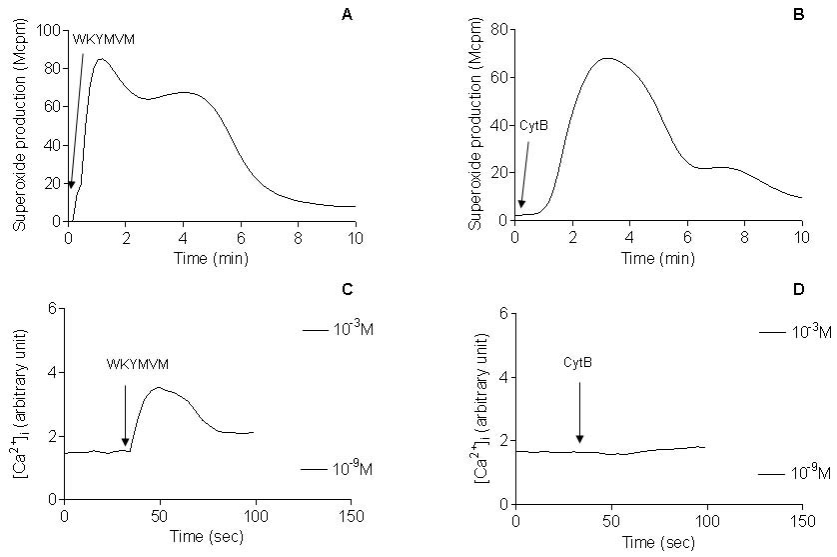
**Neutrophil superoxide anion production and intracellular calcium response induced by WKYMVM or cytochalasin B (cytB)**. Neutrophils were first incubated at 15°C for 10 min with 5 μg/ml CytB (A and C) or desensitized with 10^-7 ^M WKYMVM (B and D) followed by incubation at 37°C for another 10 minutes. The subsequent production of superoxide anion upon the addition of 10^-7 ^M WKYMVM (A) or 5 μg/ml cytB (B) (indicated by the arrows) was measured by isoluminol amplified CL technique. *Abscissa*, time of study; *ordinates*, superoxide production given as given as cpm × 10^-6^. The concentrations of intracellular calcium were determined upon stimulation with either cytB (C) or WKYMVM (D). Cells were first loaded with Fura-2 (2 μM) before pre-incubation with WKYMVM (C) or CytB (D) at 15°C. The fluorescence levels corresponding to 1 mM and 1 nM are indicated by the bars. *Abscissa*, time of study; *ordinates*, intracellular Ca^2+ ^changes given in arbitrary units. The curves are representative of at least four experiments.

The direct transfer of the system used to determine oxygen radical release to a system for determining transient rises in intracellular calcium levels revealed the expected insensitivity to PBP10 of the fMLF-induced neutrophil response (Fig 3). However, the WKYMVM-triggered response was only partly inhibited (Fig 3). This is in accordance with previously published results [28]. It is important to point out that there is no direct link between the rise in [Ca^2+^]_i _and activation of the NADPH-oxidase. This is clearly shown in desensitized cells in which the cytoskeleton is disrupted by cytochalasin B. These cells produce large quantities of oxygen radicals (Fig 4B) that is inhibited by PBP10 (data not shown), but in contrast to what is seen with control (non-desensitized) cells (Fig 4A and 4C) oxidase activation in the desensitized cells is not associated with any rise in [Ca^2+^]_i _(Fig 4D).

The sensitivity of the calcium measurement system is very low when high concentrations of agonist (10^-7 ^M) are used, and we set out to determine if the insensitivity to PBP10 of the WKYMVM induced response, is due to the concentration used. We found that when the concentration of agonist was reduced (from 10^-7 ^M, the final concentration used in Fig 3A) to 10 nM or 5 nM, the response triggered by WKYMVM was completely inhibited by PBP10 (Fig 3B). Lower concentrations of WKYMVM did not evoke any Ca^2+ ^response. In contrast to the situation observed for WKYMVM, there was no inhibition by PBP10 when the concentration of fMLF was reduced to 1 nM, which was the lowest concentration to induce a response (Fig 3B). These data clearly show that the PBP10 peptide selectively inhibits the calcium response mediated by FPR2.

### The transient increases in intracellular Ca^2+ ^induced by FPR1 and FPR2 rely on mobilization of Ca^2+ ^from intracellular storage organelles

**Figure 5:**
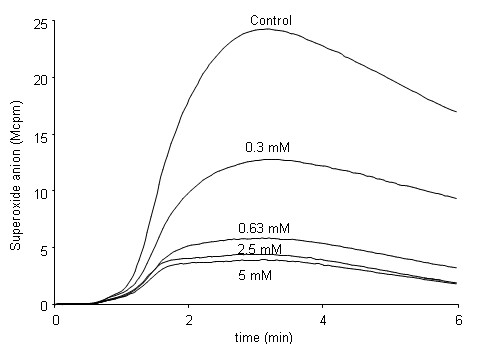
**Effects of increasing concentrations of EGTA on ionomycin-induced production of superoxide anions by neutrophils**. Neutrophils were stimulated with ionomycin (5 × 10^-7 ^M final concentration) without or with increasing concentrations of the Ca^2+ ^chelator EGTA. EGTA was added to the samples 20 seconds before the addition of the ionophore (time 0). Intracellular production of superoxide was assessed using a luminol-amplified chemiluminescence technique. Light emission was measured with or without EGTA, and a representative experiment of at least five performed is shown. *Abscissa*, time of study (minutes); *ordinate*, superoxide production, given as light emission and expressed as cpm × 10^-6^.

**Figure 6:**
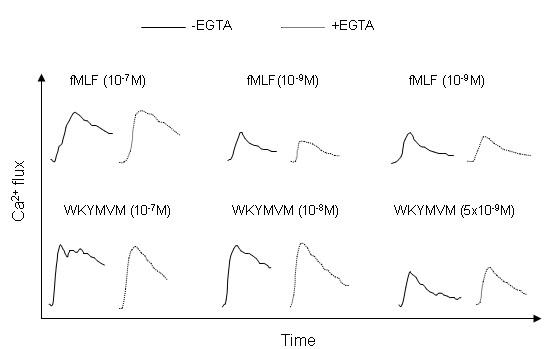
**The fMLF and WKYMVM peptides induce a rise in intracellular calcium, even when extracellular calcium is depleted**. Neutrophils loaded with Fura-2 were incubated at 37°C for 5 minutes. Depletion of extracellular calcium was achieved by the addition of EGTA (5 mM; broken lines) 20 seconds before addition of the agonist (time 0). The cells were then stimulated with decreasing concentrations of WKYMVM (lower part of the figure) or fMLF (upper part of the figure). The responses of the control cells (incubated in the absence of EGTA and in the presence of 1 mM calcium) are shown as solid lines. The changes in cytosolic Ca^2+ ^levels were determined by measuring the fluorescence emitted at 509 nm following excitation at 340 nm and 380 nm. The traces shown are from a representative experiment of at least five repetitions that gave similar results.

The signals generated by the binding of an FPR2-selective agonist to a neutrophil have been shown to induce opening of the calcium channels in the plasma membrane, without mobilization of the Ca^2+^-storing organelles [29]. Accordingly, it has been shown that there is no increase in the level of intracellular free calcium when extracellular calcium is lacking [29]. This contrasts with the signals known to be generated by the fMLF/FPR1 ligand-receptor-pairing. These signals induce the mobilization of Ca^2+ ^from intracellular storage organelles, and an increase in free intracellular calcium is also achieved in the absence of extracellular calcium (see below and [30]). To assess the effects of calcium influx on neutrophil responses, the calcium ions in the extracellular medium were removed by treatment with EGTA immediately before the addition of the receptor-specific agonist. The concentration of EGTA used was that required to achieve a maximal inhibition on ionomycin induced the NADPH-oxidase in neutrophils (Fig 5), an activity that is dependent on the influx of calcium from extracellular medium across the plasma membrane. We found that WKYMVM induced an increase in intracellular calcium in the absence of extracellular calcium (Fig 6). Similar outcomes were obtained when the FPR2 agonist MMK-1 (data not shown) or fMLF (Fig 6) was used. The kinetics of the WKYMVM-induced response resembled those of the other peptides, and we could not confirm that the Ca^2+ ^signaling induced by the FPR2 agonist induces a calcium influx across the plasma membrane that is independent of the emptying of the intracellular stores. Therefore, the pattern of responses triggered through FPR2 is identical to that mediated through FPR1.

## Discussion

Human neutrophils express the formyl peptide receptor family members FPR1 and FPR2, which share a high degree of amino acid sequence identity. Despite the similarities between the two receptors, the signaling events that follow receptor occupancy and activation differ. These differences may be related either to a specific agonist or distinct difference(s) between the receptors [4,31]. We have previously shown that a receptor agonist that binds to both FPR1 and FPR2 can trigger receptor-specific signals, and depending on the particular triggering agonist, both receptors can either inhibit the functional repertoire of the neutrophils or activate the same repertoire [32-34]. One of the early signals generated by activated neutrophil GPCRs (including FPR1 and FPR2) is the rapid rise of [Ca^2+^]_i_, which affects different cellular functions. For a resting neutrophil, the free cytosolic calcium concentration is maintained at a very low level (approximately 100 nM) relative to the level in the extracellular fluid (approximately 1 mM). To maintain this concentration gradient, cells are equipped with ion pumps that transport Ca^2+ ^out of the cells as well as to the intracellular calcium storing organelles, which contain high levels of the calcium-binding protein calreticulin. It is generally accepted that the main pathway for the control of rapid changes of [Ca^2+^]_i _in neutrophils is that represented by the capacitive model, which starts with the production of inositol-1,4,5-trisphosphate (IP_3_). The main pathway for IP_3 _production involves PLC-mediated hydrolysis of membrane PIP_2_; the generated IP_3 _releases calcium from intracellular stores by binding to specific receptors on the intracellular storage organelles. The depletion of the intracellular Ca^2+ ^stores regulates the opening of store-operated Ca^2+ ^channels (SOC) in the plasma membrane, thereby providing a rich source of capacitive entry of calcium ions, originating from the extracellular space [26,35,36]. With respect to the different signaling properties of the FPRs, a recent report has demonstrated that the regulatory mechanism that leads to an increase in [Ca^2+^]_i _is unique to FPR2. Binding of an agonist to either of the two FPRs results in a signaling cascade, which in turn leads to an increase in intracellular calcium through the influx of ions across the plasma membrane. Whereas FPR1 influences the plasma membrane channels through the emptying of intracellular stores, as described above, FPR2 appears to generate a signal that acts directly on the ion channels in the plasma membrane so as to facilitate the influx of Ca^2+ ^independently of the filling status of the storage organelles [29].

Although we could not verify this difference in signaling profile, we show that the signals from FPR1 and FPR2 initiate an increase in cytosolic free calcium derived from the emptying of intracellular stores. The increase in [Ca^2+^]_i _was achieved when the calcium in the extracellular milieu was depleted through the addition of EGTA; these data are in accordance with recent results from experiments in which a much higher concentration (10^-7 ^M) of agonist was used [37]. With respect to the different signaling properties of the two receptors, we recently showed that FPR1 and FPR2 have different sensitivities to PBP10 [28]. PBP10 inhibits FPR2-triggered activation of the neutrophil NADPH-oxidase. However, when this system was directly transferred to a calcium measurement system, we found that the cytosolic increase in free Ca^2+ ^was largely insensitive to PBP10.

In the present study, we confirm the difference between FPR1-dependent and FPR2-dependent activation of the neutrophil NADPH-oxidase. Moreover, we show that PBP10 inhibits not only the oxidase activity, but also the FPR2-induced increase in [Ca^2+^]_i_. The transient rise in [Ca^2+^]_i _peaks at agonist concentrations that are much lower than those used in the oxidase assay, and the inhibitory effects of PBP10 are, thus, disclosed only when the concentration of the FPR2 agonist is reduced. No inhibitory effect of PBP10 is seen when fMLF is used as the agonist, irrespective of the concentration of the chemoattractant used to activate the neutrophils.

The molecular mechanism underlying the effects of PBP10 on FPR2-mediated induction of cell functions is complex. PBP10 displays clearly higher specificity for FPR2 than for FPR1, although this pattern of sensitivity is not unique to this receptor [15,18]. The biochemical and biophysical characterizations of the ten strategically organized basic and hydrophobic amino acids in PBP10, which are also present in the gelsolin molecule, suggest that it may interact with a broad range of negatively charged phosphomonoesters and hydrophobic acyl chains on anionic phospholipids. This implies that in addition to its blocking/competing activities, which involve proteins that are regulated by cellular phosphoinositides, PBP10 may affect the functions of bioactive and signaling lipids. Although the precise signal transduction step that is disrupted by PBP10 remains to be elucidated, the receptor-specific and signal-selective effects of this peptide on neutrophil functions suggest that it has potential applications as a tool to manipulate and define how G-protein-coupled receptors produce and integrate the signals generated from activated receptors, as well as to probe new signaling functions of polyphosphoinositides.

## Conclusions

The neutrophil FPR family members FPR1 and FPR2 share 69% amino acid identity and mediate almost indistinguishable cellular responses. Therefore, these two FPRs use the same basic signaling pathways. Accordingly, we show that Ca^2+ ^signaling mediated *via *FPR2 follows the same route as FPR1-mediated signaling, which involves initial emptying of the intracellular stores. However, we found that the FPR2-mediated oxidase activity and Ca^2+ ^signaling pathway are inhibited by the FPR2-specific inhibitor PBP10, whereas there is no inhibitory effect of PBP10 when the high affinity FPR1 agonist fMLF is used, irrespective of the concentration of the chemoattractant used to activate the neutrophils. These data clearly demonstrate that there is a fundamental difference between the two very closely related receptor members, in that one is PBP10-sensitive (FPR2) and the other (FPR1) is PBP10-insensitive.

## Methods

### Peptides and reagents

The hexapeptide WKYMVM was synthesized and purified by Alta Bioscience (University of Birmingham, UK). The formylated peptide N-formylmethionyl-leucyl-phenylalanine (fMLF) was purchased from Sigma Chemical Co. (St. Louis, MO). The peptides were dissolved in dimethyl sulfoxide to a concentration of 10^-2 ^M, and stored at -70°C until use. Further dilutions were made in Krebs-Ringer phosphate buffer that was supplemented with glucose (10 mM), Ca^2+ ^(1 mM), and Mg^2+ ^(1.5 mM) (KRG).

### Determination of changes in cytosolic calcium

Cells at the density of 1-3 × 10^6 ^per ml were washed with Ca^2+^-free KRG and centrifuged at 220 × *g*. The cell pellets were resuspended at a density of 2 × 10^7 ^cells/ml in KRG that contained 0.1% BSA, and loaded with 2 μM Fura 2-AM (Molecular Probes, Eugene, OR) for 30 minutes at room temperature. The cells were then diluted to twice the original volume with RPMI 1640 culture medium without phenol red (PAA Laboratories GmbH, Pasching, Austria) and centrifuged. Finally, the cells were washed once with KRG, and resuspended in the same buffer at a density of 2 × 10^7 ^cells/ml. Calcium measurements were carried out in a Perkin Elmer fluorescence spectrophotometer (LC50), with excitation wavelengths of 340 nm and 380 nm, an emission wavelength of 509 nm, and slit widths of 5 nm and 10 nm, respectively. The transient rise in intracellular calcium is presented as the ratio of fluorescence intensities (340 nm: 380 nm) detected. The measuring cuvette contained catalase (2000 U), to counteract inactivation of the chemoattractants by the MPO-H_2_O_2_-system [38].

The concentration of EGTA required to achieve a calcium-free environment was determined by titration of the ionomycin-triggered production of oxidants by neutrophils resuspended in KRG that contained 1 mM Ca^2+^. A small volume (10 μl) of EGTA-containing buffer was added to the measuring vial, and 20 seconds later the cells were activated by the addition of ionomycin (5 × 10^-7 ^M final concentration). The lowest concentration of EGTA that inhibited more than 90% of the ionomycin-induced response was used in subsequent studies to ensure that no Ca^2+ ^entered the cells across the plasma membrane.

### NADPH-oxidase activity measurements

Neutrophils (5 × 10^6 ^cells/ml) were incubated at 37°C for 5 minutes in an NADPH-oxidase measuring apparatus (Berthold Co., Wildbad, Germany). The activity of the cells was then using a luminol/isoluminol-enhanced chemiluminescence (CL) system [39,40]. The extracellular CL activity was measured in a six-channel Biolumat LB 9505, using disposable polypropylene tubes with a 0.90-ml reaction mixture. The mixture contained neutrophils (5 × l0^5 ^cells/ml), horse radish peroxidase (HRP; 4U) and isoluminol (a cell-impermeable CL substrate; 2 × 10^-5 ^M). The tubes were equilibrated at 37°C for 5 minutes in the absence or presence of inhibitor, after which the stimulus (0.1 ml) was added. The light emission was recorded continuously.

To determine intracellular NADPH-oxidase activity levels, we used mixtures that contained 5 × 10^5 ^neutrophils/ml, superoxide dismutase (SOD; a cell-impermeable superoxide scavenger; 200 U), catalase (2000 U), and luminol (a cell-permeable CL substrate; 2 × 10^-5 ^M). The tubes were equilibrated at 37°C for 5 minutes in the luminometer, after which the stimulus (0.1 ml) was added, and light emission was recorded continuously.

## Authors' contributions

HF performed the experiments. HF and CD planned the project, designed the experiments, wrote the paper, and approved the final version of the paper.
